# Prospective comparison of ^18^F-PSMA-1007 PET/CT and MRI with histopathology as the reference standard for intraprostatic tumour detection and T-staging of high-risk prostate cancer

**DOI:** 10.1007/s00259-025-07208-z

**Published:** 2025-03-31

**Authors:** Aino Kivikallio, Simona Malaspina, Irena Saarinen, Marko Seppänen, Mikael Anttinen, Ivan Jambor, Janne Verho, Jukka Kemppainen, Hannu J. Aronen, Peter J. Boström, Otto Ettala, Pekka Taimen

**Affiliations:** 1 Institute of Biomedicine and Department of Pathology, University of Turku and Turku University Hospital, Turku, Finland; 2 FICAN West Cancer Centre, University of Turku and Turku University Hospital, Turku, Finland; 3 Turku PET Centre, University of Turku and Turku University Hospital, Turku, Finland; 4 Department of Urology, University of Turku and Turku University Hospital, Turku, Finland; 5 Department of Diagnostic Radiology, University of Turku and Turku University Hospital, Turku, Finland; 6 Enterprise Service Group - Radiology, Mass General Brigham and Harvard Medical School, Boston, MA USA

**Keywords:** Prostate cancer, Primary staging, Tumour staging, ^18^F-PSMA-1007 PET/CT, WBMRI, PSMA, Immunohistochemistry

## Abstract

**Purpose:**

To prospectively compare the ability of ^18^F-PSMA-1007 PET/CT and whole-body MRI (WBMRI) with DWI to detect prostate cancer (PCa) lesions and assess their local stage. Additionally, to evaluate the correlation between PSMA uptake on PET/CT and PSMA expression as assessed by immunohistochemistry.

**Methods:**

Men with newly diagnosed unfavourable intermediate or high-risk PCa underwent ^18^F-PSMA-1007 PET/CT and WBMRI with DWI before robot-assisted laparoscopic prostatectomy. Diagnostic accuracy for intraprostatic tumour localization, seminal vesicle invasion (SVI), and extraprostatic extension (EPE) was evaluated using whole-mount prostatectomy specimens as the reference standard. SUVmax was compared with immunohistochemical PSMA staining intensity quantified using QuPath software.

**Results:**

19 patients with 39 intraprostatic lesions in histopathology were included. The overall lesion detection rates for PET/CT were 84.6% and 82.1% for two independent readers, compared to 74.4% and 46.2% for MRI readers. The detection rates of index lesions were 94.7% for PET/CT and 74.0–84.0% for MRI, whereas those of non-index lesions were 70.0–75.0% for PET/CT and 20.0–65.0% for MRI. For detecting EPE, AUC values were 0.500-0.591 for PET/CT and 0.648–0.682 for MRI. For detecting SVI, AUC values ranged from 0.629 to 0.700 across both modalities. SUVmax showed a weak correlation with immunohistochemical expression of PSMA multiplied by lesion diameter (Spearman’s ρ = 0.427, *p* = 0.013). Lesion diameters measured using 30% and 40% of SUVmax, as well as prostate SUVbackground x2, showed the closest agreement with histopathological measurements.

**Conclusion:**

^18^F-PSMA-1007 PET/CT demonstrated high sensitivity in localizing intraprostatic carcinoma lesions but seemed inferior to WBMRI in detecting EPE. PSMA uptake appears to depend on both PSMA expression and lesion size. These findings highlight the complementary roles of PET/CT and MRI in the detection and tumor staging of PCa.

**Clinical trial registration:**

Clinicaltrials.gov ID: NCT03537391. Registered 25 May 2018.

**Supplementary Information:**

The online version contains supplementary material available at 10.1007/s00259-025-07208-z.

## Introduction

Prostate cancer (PCa) is the second most common cancer in men, accounting for 14.1% of new cases worldwide [1]. Accurate primary staging, essential for guiding treatment decisions, heavily relies on imaging [2]. Prostate-specific membrane antigen (PSMA) positron emission tomography/computed tomography (PET/CT) has shown superior specificity and sensitivity in detecting lymph node and bone metastases compared to conventional imaging [3], while multiparametric (mp) magnetic resonance imaging (MRI) remains the gold standard for detecting intraprostatic tumours and local staging (T-staging). However, a recent meta-analysis found PSMA PET/MRI to be superior and PSMA PET/CT comparable to mpMRI in detecting index tumours, extraprostatic extension (EPE), and seminal vesicle invasion (SVI) [4]. On the other hand, another meta-analysis reported PSMA PET/CT to be less sensitive than mpMRI in detecting EPE and SVI [5].

Despite these findings, limited research has assessed the accuracy of PSMA PET/CT in detecting non-index tumours or localizing intraprostatic lesions beyond lateralization, especially in prospective studies. Furthermore, the use of fluorine-18 PSMA-1007 (^18^F-PSMA-1007), the only PSMA tracer with minimal urinary excretion currently in clinical use, remains underexplored in the context of T-staging. This characteristic offers a distinct advantage for detecting intraprostatic disease and improving staging accuracy compared to other PSMA tracers.

PSMA is a type II membrane protein expressed in the epithelium of the prostatic gland and certain benign tissues. Although PSMA is typically overexpressed in PCa, entirely negative and heterogeneous staining patterns are also well-documented [6]. Immunohistochemical overexpression of PSMA has been associated with higher Gleason scores and other traditional prognostic factors [7, 8]. Nonetheless, the few studies investigating the correlation between PSMA uptake on PET and PSMA expression on immunohistochemistry (IHC) have produced inconsistent results.

The aims of the current study were, first, to compare the diagnostic performance of ^18^F-PSMA-1007 PET/CT and whole-body MRI (WBMRI) for T-staging and detection of intraprostatic lesions using whole-mount prostatectomy specimens as the reference standard, and second, to evaluate the potential correlation between PSMA uptake on PET/CT and the immunohistochemical PSMA staining intensity in PCa lesions.

## Materials and methods

### Study design and patient population

This single-centre, prospective, non-randomized study, (PROSTAGE, registered as NCT03537391) included patients with newly diagnosed PCa of unfavourable intermediate- to high-risk (defined as International Society of Urological Pathology grade group [ISUP GG] ≥ 3, PSA ≥ 20 ng/ml, and/or clinical stage cT ≥ T3). The primary aim of the study was to compare ^18^F-PSMA-1007 PET/CT, WBMRI with diffusion-weighted imaging (DWI) and conventional imaging modalities – including ^99m^Tc-hydroxymethylene diphosphonate bone scintigraphy, single-photon emission computed tomography-CT (SPECT-CT), and contrast-enhanced CT of the thorax, abdomen and pelvis – for the overall staging of PCa.

The results of nodal (N-staging) and distant metastasis (M-staging) using these imaging modalities have already been reported [9, 10]. This report focuses on intraprostatic tumor detection and T-staging in patients who underwent robot-assisted laparoscopic prostatectomy (RALP).

The key exclusion criteria – prior imaging for PCa metastasis staging or contraindication to MRI – were consistent with those of the PROSTAGE study. For this analysis, an additional exclusion criterion was applied: previous PCa treatment before prostatectomy. All participants underwent WBMRI with DWI and ^18^F-PSMA-1007 PET/CT prior to RALP. The study was conducted in accordance with the principles of the Declaration of Helsinki and was approved by the Ethics committee of the Hospital District of Southwest Finland.

### Imaging modalities

The PET/CT scans were performed using the Discovery MI digital PET/CT system from GE Healthcare (Milwaukee, WI, USA). For attenuation correction, a low-dose CT protocol was used for the transmission scan, featuring a noise index of 30, automatic 3D current modulation (10 to 120 mAs) and 120 kVp. A static emission scan was conducted from the vertex to mid-thigh, covering six bed positions with an acquisition time of two minutes per bed. The sinogram data were corrected for deadtime, decay, and photon attenuation, and reconstructed into a 256 × 256 matrix. Image reconstruction utilized the Q. Clear method, a Bayesian penalized likelihood algorithm for PET, with a β value of 500, incorporating random and scatter corrections. The final in-plane resolution, measured as full-width half-maximum (FWHM), was less than 5 mm.

The WBMRI scans were performed using the Siemens Avanto fit 1.5 T MR system (Siemens Healthcare GmbH, Erlangen, Germany). The imaging protocol consisted of axial T2-weighted fat suppressed (FS) half-Fourier single-shot turbo spin-echo images (HASTE), axial short-tau inversion recovery (STIR) DWI with *b*-values of 0, 50 and 900 s/mm^2^, and coronal 3D T1-weighted volumetric interpolated breath-hold examination (VIBE) Dixon sequences. Additionally, STIR DWI images from the level of the pelvis with *b*-values 0 and 1500 s/mm2 were included.

### Imaging interpretation and analysis

PSMA PET/CT and WBMRI images were independently reviewed by two nuclear medicine physicians (8 and 6 years of experience in PSMA PET/CT) and radiologists (12 and 9 years of experience in prostate MRI), respectively. Interpretations were based on clinical expertise and current guidelines [11, 12]. All readers were aware of the diagnosis of PCa but blinded to other clinical, imaging and pathological data.

A PCa-positive lesion on PSMA PET/CT was defined as a focal uptake with activity at least twice that of surrounding prostate tissue, while diffuse and homogeneous increased uptake was not considered PCa-positive. Maximum standardized uptake value (SUVmax) was measured for each lesion. An additional volumetric analysis was performed on index lesions, with a manually defined constraint limited to the prostate gland. Thresholds of 20%, 30%, 40%, and 50% of SUVmax, as well as prostate SUVbackground ×1 and prostate SUVbackground ×2, were assessed. The maximum transaxial diameter of the generated tumour volume was measured for each threshold (Supplementary Figure S1) and compared to the maximum transaxial diameter measured in histology. In cases where PCa affected the entire prostate gland (*n* = 2), the average prostate background measured from other patients was used as a reference.

In WBMRI, lesions with a score of 3 or higher on the Prostate Imaging Reporting and Data System (PI-RADS) were considered positive for PCa. 

In addition to lesion localization, the presence of EPE and SVI were evaluated at the lesion level. In WBMRI, the likelihood of EPE was assessed using a five-point scale, with scores of 4 and 5 regarded as positive for EPE.

### Histopathological analysis

The prostatectomy specimens were processed and evaluated following current guidelines [2, 13]. After formalin fixation, the specimens were dissected at 5 mm intervals and embedded in paraffin as whole-mounts. Histological sections, 4 μm thick, were prepared for routine haematoxylin and eosin (H&E) staining and IHC. Lab Vision autostainer (Thermo Fisher Scientific) was used for PSMA staining with a mouse monoclonal PSMA antibody (Dako, clone M3620, 1:100). The slides were digitized with Pannoramic 1000 slide scanner (3DHistec, Hungary) at 20x resolution and analysed with the open-source software QuPath (v. 0.5.0) [14]. Histopathological evaluation was conducted under the supervision of a dedicated uropathologist who was blinded to imaging data. Only PCa lesions with a diameter ≥ 3 mm were included in the analyses. Anatomical location, maximum transaxial diameter, ISUP GG and the presence of EPE, SVI, extensive perineural invasion (defined as > 10 instances with some extending beyond the tumour bulk), ductal carcinoma and cribriform growth pattern were assessed for each lesion. The previously reported 4.3% linear shrinkage of prostatectomy specimens during tissue processing was considered marginal and not taken into account in measuring lesion diameters [15]. The lesion with the highest GG and, in case of multiple lesions with identical GG, the lesion larger in diameter was selected as the index lesion. For IHC slide analysis, lesions were manually annotated, and a cell detection tool was applied to the annotated areas. A supervised object classifier, trained on manually labelled data, was used to identify PCa cells. The software calculated the maximum optical density (ODmax) of cytoplasmic 3,3’-diaminobenzidine (DAB) staining for each identified PCa cell. The mean ODmax was reported for each lesion and for a manually selected hotspot of 20,000 (± 1,000) cells with the strongest staining. “Mean ODmax of cytoplasmic DAB” refers to the per-lesion mean value if not stated otherwise.

### Segment-level analysis in imaging and histopathology

To evaluate the anatomical location of each lesion in both imaging and histopathology, the prostate was divided into 12 segments. The basis, mid gland and apex were each subdivided into quadrants: right/left and anterior/posterior. Each of the 12 segments was classified as either positive or negative for the presence of a lesion. Lesions identified by imaging and histopathology were considered to match if they overlapped in at least one segment.

### Consensus reading

Following segment-level analysis, a consensus reading was conducted between the pathologist and PET reader 2 to resolve discrepancies in the interpretation of anatomical locations. The pairing of PCa lesions identified in histopathology and PET was manually reviewed case by case and, if multiple PET lesions corresponded to a single histopathological lesion or vice versa, the lesions were grouped accordingly. False-positive PET lesions were re-evaluated in histopathology, and the corresponding tissue blocks were stained for PSMA.

### Statistical analysis

Descriptive statistics for categorical variables were reported as frequency and percentage, while continuous variables were summarized using median and interquartile range (IQR). Detection rates were compared between imaging modalities using Pearson’s Chi-squared test with Yate’s continuity correction. Sensitivity, specificity and accuracy were reported with 95% confidence intervals (CI) and compared between modalities using Fisher’s exact test. The area under the curve (AUC) was calculated with 95% CI and compared between modalities using DeLong’s test. Student’s t-test was employed to compare the characteristics of index and non-index lesions, with Levene’s test used to assess equality of variances. Interobserver agreement was evaluated using Cohen’s Kappa. Correlations between SUVmax, ODmax, and ISUP GG were analyzed using Spearman’s rank correlation coefficient. Normality was assessed using the Shapiro-Wilk test. A one-sample Wilcoxon test was used to analyse the difference between lesion diameters in histology and PET/CT. A *p*-value of less than 0.05 was considered statistically significant. Statistical analyses were performed using IBM SPSS Statistics (version 29.0.0.0) for Windows and R (version 3.6.3).

## Results

A total of 19 biopsy-proven PCa patients underwent ^18^F-PSMA-1007 PET/CT, WBMRI with DWI, and RALP. The patient characteristics are summarized in Table 1. The median age of the cohort was 65 years (IQR = 61.5–68.5), and the median PSA level was 9.7 ng/ml (IQR = 6.5–13.5). Pathological staging (pT) revealed that 42% of patients had pT2 disease, 32% had pT3a, and 26% had pT3b. The ISUP GG ranged from 2 to 5, with the majority (84%) classified as ISUP GG 3–5.

**Table 1:** Patient and disease characteristics (*n* = 19)

**Age (yr), median (IQR)**	65 (61.5, 68.5)
**Blood PSA level (ug/l)**,** median (IQR)**	9.7 (6.5, 13.5)
**Clinical T-stage**	
cT1	3 (16%)
cT2	13 (68%)
cT3	3 (16%)
**Pathological T-stage**	
pT2	8 (42%)
pT3a	6 (32%)
pT3b	5 (26%)
**Pathological N-stage**	
pN0	10 (67%)
pN1	5 (33%)
Unknown	4
**ISUP grade group**	
2	3 (16%)
3	10 (53%)
4	1 (5%)
5	5 (26%)

yr = year, PSA = prostate-specific antigen, IQR = interquartile range

A total of 39 intraprostatic lesions were identified through histopathological analysis. The histopathological characteristics of index and non-index lesions are summarized in Table 2. Index lesions were larger in diameter and exhibited more cribriform growth pattern and extensive perineural invasion compared to non-index lesions. Two index lesions also demonstrated a ductal carcinoma component in addition to acinar carcinoma. Immunohistochemical PSMA expression, measured as ODmax of cytoplasmic DAB, was significantly higher in index lesions than in non-index lesions (*p* = 0.006).

**Table 2:** Histopathological characteristics of index and non-index lesions

	Index lesions (*n* = 19)	Non-index lesions (*n* = 20)
**Maximum diameter (mm)**,** median (IQR)**	17 (11.0, 24.0)	10 (6.3, 15.8)
**ISUP grade group**,** n (%)**		
1	-	7 (35%)
2	2 (11%)	11 (55%)
3	6 (32%)	2 (10%)
4	5 (26%)	-
5	6 (32%)	-
**Cribriform pattern**,** n (%)**		
0%	3 (16%)	17 (85%)
1–25%	8 (42%)	3 (15%)
26–50%	2 (11%)	-
51–75%	4 (21%)	-
76–100%	2 (11%)	-
**Extensive perineural invasion**,** n (%)**	11 (58%)	1 (5%)
**ODmax of cytoplasmic DAB**,** median (IQR)**	1.227 (0.772, 1.962)	0.639 (0.326, 1.186) ^a^

^a^ Statistically significant difference (*p* < 0.05) compared to index lesions

IQR = interquartile range, ODmax = maximum optical density, DAB = 3,3’-diaminobenzidine

The lesion-based detection rates for both imaging modalities are presented in Table 3. PSMA PET/CT showed high sensitivity for both index lesions (94.7%) and non-index lesions (70.0–75.0%). Interobserver agreement between PET readers was substantial (κ = 0.759). WBMRI, in contrast, showed lower sensitivity for both index lesions (74.0–84.0%) and non-index lesions (20.0–65.0%). Interobserver agreement between MRI readers was low (κ = 0.189). A statistically significant difference was observed between MRI reader 2 and both PET readers in detecting all lesions and non-index lesions (*p* < 0.01). False-positive lesions were identified by both imaging modalities, with counts of 4 and 4 for PET readers 1 and 2, respectively, and 7 and 1 for MRI readers 1 and 2, respectively.

**Table 3:** Detection rates of PSMA PET/CT and WBMRI with DWI for all lesions, index lesions and non-index lesions

	PSMA PET/CT reader 1	PSMA PET/CT reader 2	MRI reader 1	MRI reader 2
**All lesions (*n*** **= 39)**				
DR	33 (84.6%)	32 (82.1%)	29 (74.4%)	18 (46.2%) ^a, b^
**Index lesions (***n* **= 19)**				
DR	18 (94.7%)	18 (94.7%)	16 (84.0%)	14 (74.0%)
**Non-index lesions (***n* **= 20)**				
DR	15 (75.0%)	14 (70.0%)	13 (65.0%)	4 (20.0%) ^a, b^

^a^ Statistically significant difference (*p* < 0.05) compared to PSMA PET/CT reader 1

^b^ Statistically significant difference (*p* < 0.05) compared to PSMA PET/CT reader 2

DR = detection rate = sensitivity

The patient-level sensitivity, specificity, accuracy and AUC values for detecting EPE and SVI are summarized in Table 4. PET/CT showed poor sensitivity but excellent specificity for detecting EPE (sensitivity: 0.182 and specificity: 1.000 for PET reader 1). PET reader 2 reported all patients as negative for EPE. WBMRI demonstrated slightly better sensitivity and good specificity (sensitivity: 0.364–0.545; specificity: 0.750-1.000) for detecting EPE. However, the only statistically significant differences were observed in sensitivity between PET/CT reader 1 and MRI reader 1 (*p* = 0.012) and in AUC between PET/CT reader 2 and MRI reader 2 (*p* = 0.017). AUC values for EPE detection were 0.500-0.591 for PET and 0.648–0.682 for MRI. Agreement between MRI readers was fair (κ = 0.305).

For detecting SVI, both imaging modalities showed similar sensitivity and specificity (sensitivity: 0.400; specificity: 0.857-1.000). AUC values for all four readers ranged from 0.629 to 0.700. PET readers showed substantial interobserver agreement for detecting SVI (κ = 0.612), whereas the agreement between the MRI readers was moderate (κ = 0.441). Supplementary Figure S2 illustrates the only case of SVI that was accurately detected by MRI but not identified by PET/CT.

**Table 4:** Diagnostic accuracy of PSMA PET and WBMRI with DWI in detecting extraprostatic extension and seminal vesicle invasion (*n* = 19)

	PSMA PET/CT reader 1	PSMA PET/CT reader 2	MRI reader 1	MRI reader 2	
**Extraprostatic extension**					
Sensitivity (95% CI)	0.182 (0.032–0.522)	0.000 (0.000-0.321)	0.545 (0.246–0.819) ^a^		0.364 (0.124–0.684)
Specificity (95% CI)	1.000 (0.598-1.000)	1.000 (0.598-1.000)	0.750 (0.356–0.955)		1.000 (0.598-1.000)
Accuracy (95% CI)	0.526 (0.293–0.748)	0.727 (0.393–0.927)	0.632 (0.386–0.828)		0.632 (0.386–0.828)
AUC (95% CI)	0.591 (0.471–0.710)	0.500 (0.500–0.500)	0.648 (0.425–0.870)		0.682 (0.533–0.831) ^b^
**Seminal vesicle invasion**					
Sensitivity (95% CI)	0.400 (0.073–0.830)	0.400 (0.073–0.830)	0.400 (0.073–0.830)		0.400 (0.073–0.830)
Specificity (95% CI)	0.857 (0.562–0.975)	1.000 (0.732-1.000)	1.000 (0.732-1.000)		1.000 (0.732-1.000)
Accuracy (95% CI)	0.737 (0.486–0.899)	0.842 (0.595–0.958)	0.842 (0.595–0.958)		0.842 (0.595–0.958)
AUC (95% CI)	0.629 (0.370–0.887)	0.700 (0.460–0.940)	0.700 (0.460–0.940)		0.700 (0.460–0.940)

^a^ Statistically significant difference (*p <* 0.05) compared to PSMA PET/CT reader 1

^b^ Statistically significant difference (*p <* 0.05) compared to PSMA PET/CT reader 2

AUC = area under the curve

After the consensus reading between PET/CT and histopathology, six false-negative and two false-positive lesions remained. The false-positive lesions showed nodular hyperplasia with heterogenous PSMA expression similar to that of the surrounding benign prostate tissue. The six false-negative lesions, all non-index lesions, are summarized in Table 5. These lesions were classified as ISUP GG 1 or 2, lacked cribriform growth, and exhibited weak PSMA staining intensity (median ODmax of cytoplasmic DAB median 0.297, IQR 0.269–0.526). Detailed characteristics of all the histological lesions and the two false-positive lesions detected by PSMA PET/CT reader 2, paired according to the consensus reading, are given in Supplementary Table S1. Histological and PSMA PET/CT images of all the false-negative and false-positive lesions are presented in Supplementary Figures S3 and S4, respectively.

**Table 5:** Characteristics of the false-negative lesions of PSMA PET/CT after consensus reading (*n* = 6)

**Maximum diameter (mm), median (IQR)**	9.5 (6.25–15.50)
**ODmax of cytoplasmic DAB**,** median (IQR)**	0.297 (0.269–0.526)
**ISUP grade group**,** n (%)**	
1	4 (67%)
2	2 (33%)
**Cribriform pattern**,** n (%)**	0 (0%)
**Extensive perineural invasion**,** n (%)**	0 (0%)
**Ductal carcinoma**,** n (%)**	0 (0%)

IQR = interquartile range, ODmax = maximum optical density, DAB = 3,3’-diaminobenzidine

**Fig. 1:**
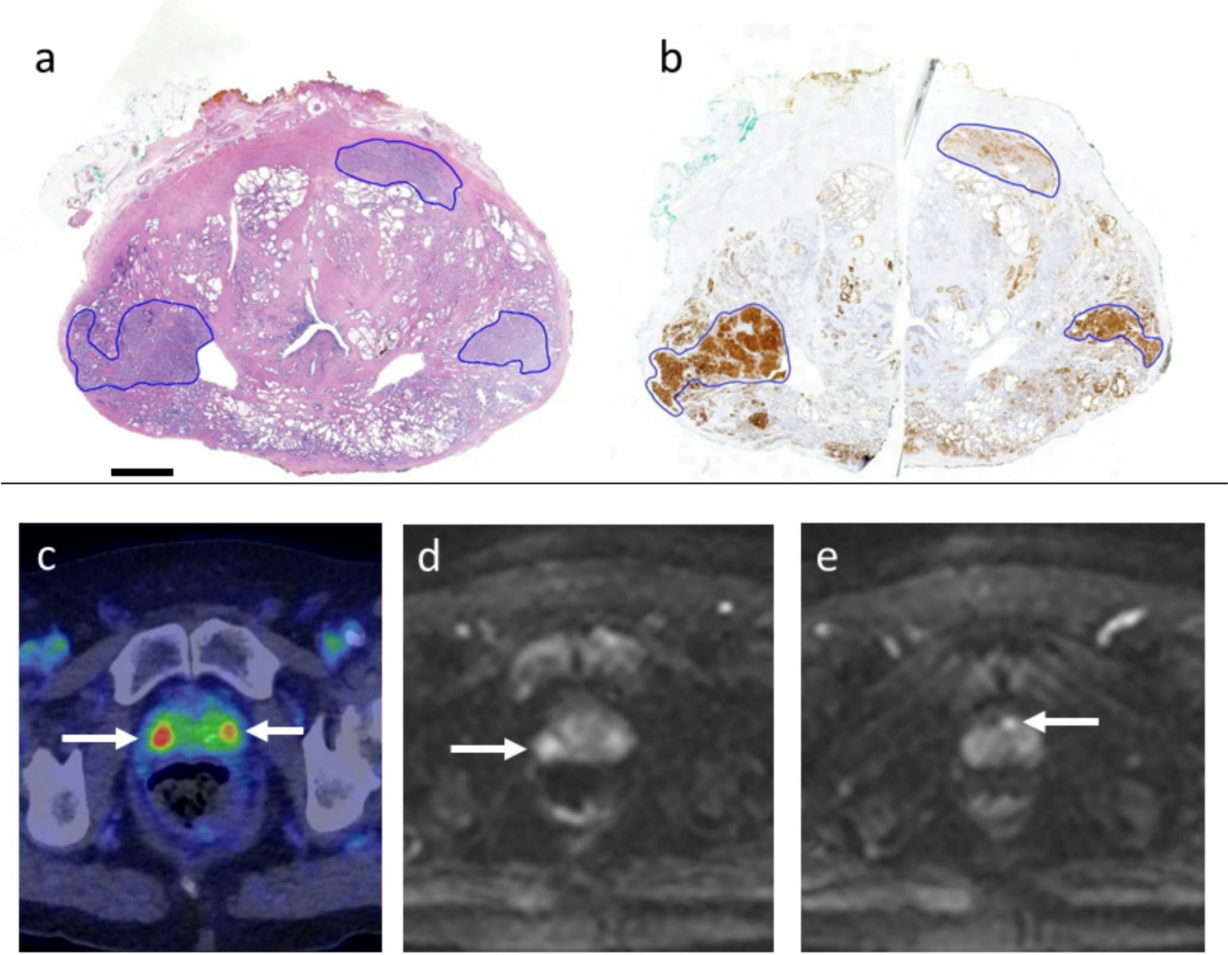
PET/CT accurately detected intraprostatic tumours with strong PSMA staining. (**a**) HE-stained cross section of mid gland, low magnification. Three PCa lesions are present: right posterior (index lesion, Gleason 4 + 4), left posterior (Gleason 4 + 3) and left anterior (Gleason 3 + 4). (**b**) Immunohistochemical PSMA staining, low magnification. The right and left posterior lesions show strong staining intensity (mean DAB ODmax 1.164 and 1.838), while the left anterior lesion shows weak to moderate staining intensity (mean DAB ODmax 0.241). (**c**) Fused PET/CT images of the prostate show two pathological uptakes (arrows), one on the right posterior mid gland (SUVmax 9,6) and the other on the left posterior mid gland (SUVmax 5,8). These lesions correspond to the Gleason 4 + 4 and 4 + 3 lesions in histopathology. (**d-e**) WBMRI DWI shows diffusion restriction in the right posterior and left anterior lesion (arrows), while the left posterior lesion is not confidently visualised. Scale bar 5 mm (**a-b**)

**Fig. 2:**
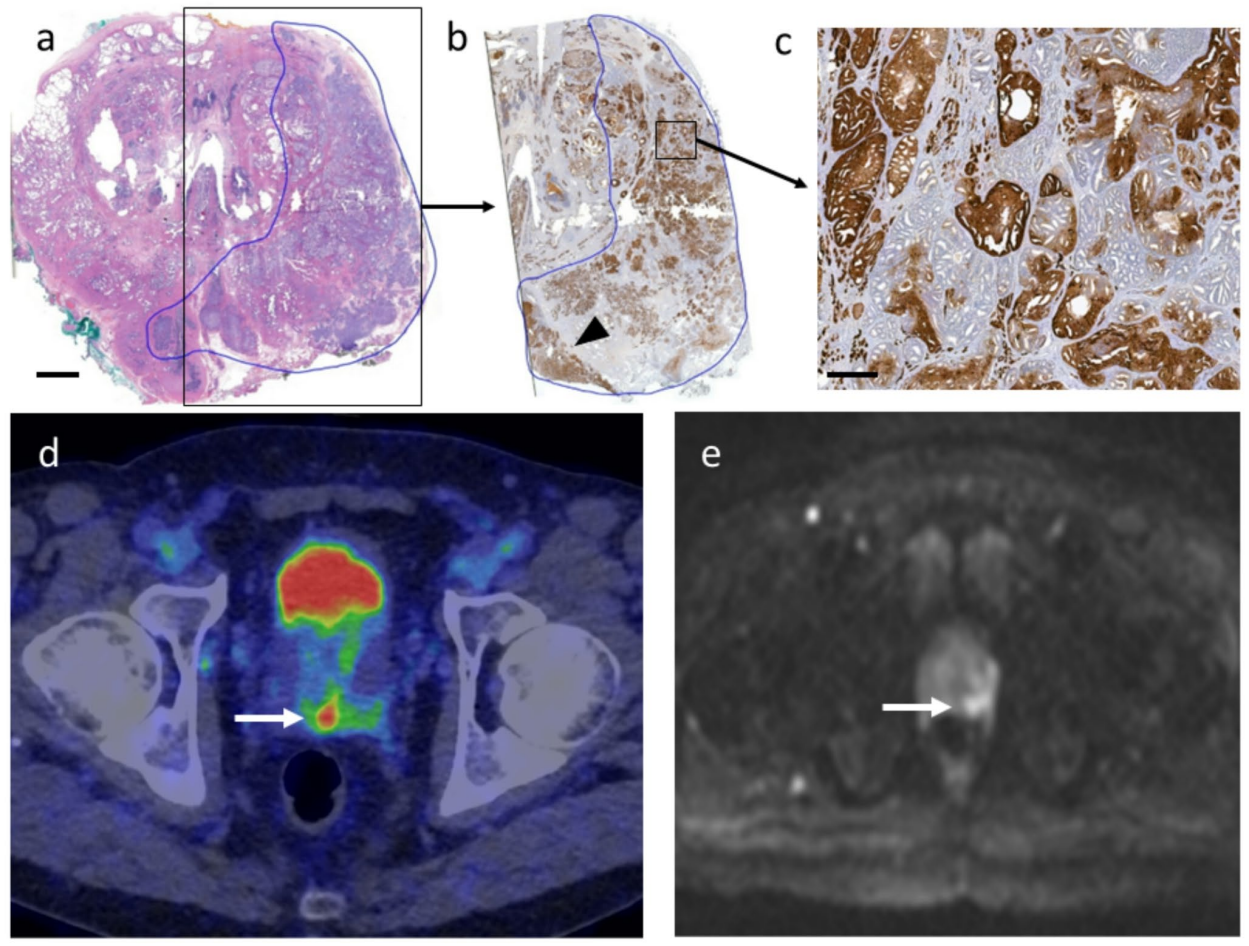
Heterogenous PSMA staining leading to the underestimation of lesion size in PET/CT. (**a**) HE-stained cross section of mid gland, low magnification. A large index lesion (Gleason 4 + 5) covers the left lobe and shows extensive EPE as well as bilateral SVI. (**b**) Immunohistochemical PSMA staining, low magnification. PSMA staining intensity is heterogeneous. More homogenous, strong PSMA expression is seen around and in the left seminal vesicle (arrowhead). (**c**) Immunohistochemical PSMA staining, high magnification. Abundant cribriform growth pattern is seen. PSMA staining is notably heterogenic with completely negative glands mixed with highly positive glands. (**d**) Fused PET/CT images show pathological uptake (arrow) in the posterior base and left seminal vesicle (SUVmax 7.0). The remaining lesion of the left lobe, the IHC staining of which was heterogenous, does not show clear pathological uptake. (**e**) WBMRI DWI shows diffusion restriction in the lesion in the left mid gland and base (arrow). EPE and SVI was detected on the left. Scale bars 5 mm (**a-b**) and 0.5 mm (**c**)

In true-positive lesions, SUVmax showed a weak but statistically significant correlation with the mean per-lesion ODmax multiplied by lesion diameter (Spearman’s ρ = 0.427, *p* = 0.013; Supplementary Figure S5). However, no significant correlation was observed between SUVmax and the mean ODmax of entire lesions or 20,000-cell hotspots (*p* = 0.214 and 0.195, respectively), nor between SUVmax and lesion diameter (*p* = 0.184) alone. Both SUVmax and mean per-lesion ODmax demonstrated significant correlations with ISUP GG (Spearman’s ρ = 0.471 and 0.512, *p* = 0.006 and *p* < 0.001, respectively). PET/CT accurately detected tumours with strong PSMA staining intensity, as demonstrated in Fig. 1. Figure 2 highlights how heterogeneous intratumoral PSMA expression can lead to the underestimation of lesion size in PET imaging. Immunohistochemical synaptophysin staining was negative in the lesion, excluding the possibility of neuroendocrine differentiation in this specific case (data not shown).

Finally, the accuracy of different SUV thresholds in lesion volume delineation on PSMA PET was evaluated based on how well they matched the lesion diameter measured by histopathology. The results indicated that lesion diameters measured using 30% and 40% of SUVmax, as well as prostate SUVbackground ×2, did not significantly differ from the histological diameter (*p*-values 0.379, 0.313, and 0.099, respectively). The smallest percentual difference between the diameters was observed with prostate SUVbackground ×2 (median 0%, IQR − 44%, 7%), 30% of SUVmax (median 7%, IQR − 4%, 17%), and 40% of SUVmax (median − 8%, IQR − 21%, 3%). The data and results of the diameter analysis are presented in Table 6.

**Table 6:** The comparison of index lesion diameters measured in histopathology and PSMA PET/CT. Six different thresholds were applied in PET/CT (20%, 30%, 40% and 50% of SUVmax, prostate SUVbackground x1 and x2). A missing value (N/A) indicates that the lesion could not be evaluated at the specific threshold

Patient	Histological diameter (mm)	SUVmax	Diameter in PET/CT using different thresholds					
			20% of SUVmax	30% of SUVmax	40% of SUVmax	50% of SUVmax	SUVbackground *1	SUVbackground *2
1	13	24.9	16	14	12	10	18	14
2	11	51.7	7.5	6.5	5.3	5.0	26	11
3	6.0	14.3	N/A	12	10	8.0	N/A	10
4	14	7.6	N/A	N/A	14	11	N/A	15
5	13	22.0	15	14	11	10	16	14
6	9.0	6.3	N/A	N/A	N/A	N/A	N/A	5.0
7	11	9.6	N/A	17	14	11	N/A	11
8	20	8.5	N/A	N/A	25	21	21	8.0
9	19	12	N/A	19	17	15	N/A	17
10	21	43.2	20	17	14	13	24	21
11	24	22.2	23	22	19	17	26	20
12	39	7.0	55	48	31	21	20	4.4
13	24	20.7	27	24	22	19	25	20
14	37	15.7	44	40	38	36	44	39
15	17	27.1	20	18	17	16	24	20
16	14	18.8	25	16	11	8.9	27	11
17	10	15.9	20	12	12	8.0	17	10
18	42	7.1	51	45	43	40	40	10
19	18	19.1	23	5.6	4.7	5.0	24	6.0
**Difference between diameters (%)**		**Median (IQR)**	19 (13, 28)	7 (-4, 17)	-8 (-21, 3)	-21 (-36, -5)	21 (5, 41)	0 (-44, 7)
		**Min**,** max**	-32, 100	-69, 100	-74, 67	-72, 33	-49, 136	-89, 67
**One-sample Wilcoxon test**, *p***-value**			0.021	0.379	0.313	0.004	0.011	0.099

SUVmax = maximum standardized uptake value, IQR = interquartile range

## Discussion

This prospective clinical study compared the diagnostic performance of ^18^F-PSMA-1007 PET/CT and WBMRI with DWI in the detection and T-staging of PCa, using histopathology from RALP as the reference. PSMA PET/CT demonstrated higher detection rates for intraprostatic lesions compared to WBMRI, although the differences were mostly statistically insignificant. Both modalities showed similar performance in detecting SVI, while WBMRI appeared slightly better at detecting EPE. However, these differences were predominantly statistically insignificant. SUVmax showed a weak but statistically significant correlation with the mean per-lesion ODmax multiplied by lesion diameter (Spearman’s ρ = 0.427, *p* = 0.013).

Several previous studies have reported the high sensitivity of ^18^F-PSMA-1007 PET/CT for detecting index lesions, with sensitivities ranging from 94 to 100% [16–19]. Our findings align with this, as PET/CT demonstrated a sensitivity of 94.7% for index lesions.

In detecting and localizing both index and non-index lesions, results in prior studies have varied [18–21]. Sensitivity has been reported as high as 95% when limited to clinically significant PCa [21] and as low as 46.6% when including smaller lesions without diameter criteria [19]. In our study, the sensitivity of PSMA PET/CT in detecting all lesions was as high as 82.1–84.6%. Notably, our analysis included lesions ≥ 3 mm in diameter, irrespective of ISUP GG, and avoided the ‘neighbouring’ approach, which is often used to account for segmentation discrepancies. Our findings further highlight the role of PSMA expression and ISUP GG in influencing detection, as false-negative PET/CT lesions in our study were all non-index lesions with ISUP GG 1–2 and low PSMA expression.

In our study, a PCa-positive lesion on PSMA PET/CT was defined as focal uptake with activity at least twice that of the surrounding prostate tissue. The two independent readers achieved high inter-reader agreement and detection rates. However, several grading systems have been proposed to enhance the accuracy of PSMA PET/CT in detecting clinically significant PCa, including the PRIMARY score [22]. The PRIMARY score incorporates both the pattern and intensity of PSMA uptake to determine the likelihood of PCa on a five-point scale. It has been shown to surpass PI-RADS for mpMRI in interobserver agreement while demonstrating comparable diagnostic performance [23]. Regarding false positives and negatives in our study, the false positive case 17 (Supplementary Fig. S4) would have been correctly classified if the PRIMARY score had been applied, considering the uptake pattern.

The performance of ^18^F-PSMA-1007 PET/CT and MRI in detecting EPE has also been inconsistent in prior studies. While some studies have found MRI superior in detecting EPE [24], others have shown PET/CT to be statistically superior in determining the accurate pT-stage and identifying EPE [18]. Similarly, the detection of SVI has shown varied results. Some studies suggest that PET/CT offers better sensitivity for SVI [24], while others have reported no significant difference between PET/CT and MRI [18, 21].

In our study, both imaging modalities demonstrated low sensitivity and high specificity for detecting EPE and SVI, with no major differences in overall performance (AUC values ≤ 0.700). Although the differences were largely statistically insignificant, MRI seemed to perform slightly better in detecting EPE, while both modalities performed nearly identically in detecting SVI. These findings may reflect the limited spatial resolution of PET for identifying the prostatic capsule and the insufficient soft tissue contrast of CT.

The few studies investigating the correlation between PSMA expression in IHC and PSMA uptake in PET imaging have produced somewhat conflicting results. Some studies have demonstrated a correlation between SUVmax and either PSMA immunoreactivity score [25, 26], or the percentage of positive cells only [27]. Another study demonstrated lower SUVmax to be associated with larger PSMA-negative tumour area but reported no correlation between SUVmax and PSMA expression in IHC [28]. The only study to use quantitative IHC analysis somewhat similar to ours also demonstrated no statistically significant correlation between SUVmax and IHC [29]. Some studies have suggested an association between tumour size and SUVmax [27, 28], while others reported no correlation between the two [25, 26]. Our study demonstrated a statistically significant correlation between ^18^F-PSMA-1007 PET uptake and quantitatively evaluated immunohistochemical staining intensity of PSMA, with the latter being multiplied by lesion diameter. SUVmax did not correlate with either per-lesion or per-hotspot mean ODmax, or lesion diameter alone, suggesting that both PSMA expression level and lesion size contribute to SUVmax. Both SUVmax and per-lesion mean ODmax correlated with the ISUP GG, a finding consistent with previous studies [26, 28].

Accurate tumour volume evaluation is essential for planning targeted radiotherapy; however, no consensus exists on the optimal methodology for tumour delineation in PSMA PET/CT [30]. Two studies using ^18^F-PSMA-1007 PET/CT have assessed the optimal SUV threshold for volume evaluation based on histopathology as the reference standard, but their findings are conflicting; one suggesting 20% and the other 42% of SUVmax [31, 32]. In our analysis, lesion diameters measured using 30% and 40% of SUVmax, as well as prostate SUVbackground ×2, showed the closest agreement with histopathological measurements. However, percentage differences between diameters remained substantial, ranging from 74 to 100%, even with the best-performing thresholds. Two key factors contributed to this variability: first, and most notably, lesions with low SUVmax (< 10 g/ml); and second, large lesions and the challenge of accurately determining background SUV.

Our study has limitations, including a small sample size, which may have reduced the statistical power to detect significant differences between modalities in T-staging. Additionally, we used WBMRI rather than mpMRI, which is the gold standard for intraprostatic cancer evaluation. Despite this, all scans were reviewed by two blinded specialists per modality, with substantial interobserver agreement for PET/CT. Discrepancies were resolved through consensus reading, ensuring accurate lesion matching without relying on the ‘neighbouring’ approach, thereby enhancing the precision of localization analyses. A major strength of our study is the use of quantitative image analysis for IHC, which reduces observer bias compared to visual assessments of staining intensity. We also extended our analysis beyond index lesions or lateralization alone, capturing a heterogeneous group of lesions ranging from ISUP GG 1 to 5.

## Conclusions

This prospective trial demonstrated the high sensitivity of ^18^F-PSMA-1007 PET/CT in detecting and localizing intraprostatic PCa lesions. PSMA uptake in PET showed a statistically significant correlation with IHC PSMA expression multiplied by lesion size. Lesion diameters measured on PSMA PET using 30% and 40% of SUVmax, as well as prostate SUVbackground ×2 thresholds, showed the closest agreement with histopathological measurements. Further research is needed to confirm the potential differences between ^18^F-PSMA-1007 PET/CT and MRI in T-staging.

## Electronic supplementary material

Below is the link to the electronic supplementary material.

Supplementary Material 1

## Data Availability

The datasets generated and analysed during the current study are available from the corresponding author on reasonable request.

## References

[1] Sung H, Ferlay J, Siegel RL, Laversanne M, Soerjomataram I, Jemal A, et al. Global cancer statistics. 2020: GLOBOCAN estimates of incidence and mortality worldwide for 36 cancers in 185 countries. CA Cancer J Clin. 2021. 10.3322/CAAC.21660 10.3322/caac.21660 33538338

[2] Cornford P, van den Bergh RCN, Briers E, Van den Broeck T, Brunckhorst O, Darraugh J, et al. EAU-EANM-ESTRO-ESUR-ISUP-SIOG guidelines on prostate cancer-2024 update. Part I: screening, diagnosis, and local treatment with curative intent. Eur Urol. 2024. 10.1016/j.eururo.2024.03.027 10.1016/j.eururo.2024.03.027 38614820

[3] Hofman MS, Lawrentschuk N, Francis RJ, Tang C, Vela I, Thomas P, et al. Prostate-specific membrane antigen PET-CT in patients with high-risk prostate cancer before curative-intent surgery or radiotherapy (proPSMA): a prospective, randomised, multicentre study. The Lancet. 2020. 10.1016/s0140-6736(20)30314-7 10.1016/S0140-6736(20)30314-7 32209449

[4] Gossili F, Mogensen AW, Konnerup TC, Bouchelouche K, Alberts I, Afshar-Oromieh A, et al. The diagnostic accuracy of radiolabeled PSMA-ligand PET for tumour staging in newly diagnosed prostate cancer patients compared to histopathology: a systematic review and meta-analysis. Eur J Nucl Med Mol Imaging. 2023. 10.1007/S00259-023-06392-0 10.1007/s00259-023-06392-0 37597010

[5] Chow KM, So WZ, Lee HJ, Lee A, Yap DWT, Takwoingi Y, et al. Head-to-head comparison of the diagnostic accuracy of prostate-specific membrane antigen positron emission tomography and conventional imaging modalities for initial staging of intermediate- to high-risk prostate cancer: a systematic review and meta-analysis. Eur Urol. 2023. 10.1016/J.EURURO.2023.03.001 10.1016/j.eururo.2023.03.001 37032189

[6] Silver DA, Pellicer I, Fair WR, Heston WDW, Cordon-Cardo C. Prostate-specific membrane antigen expression in normal and malignant human tissues. Clin Cancer Res. 1997;3(1):81–5. 9815541

[7] Bravaccini S, Puccetti M, Bocchini M, Ravaioli S, Celli M, Scarpi E, et al. PSMA expression: a potential ally for the pathologist in prostate cancer diagnosis. Sci Rep. 2018. 10.1038/S41598-018-22594-1 10.1038/s41598-018-22594-1 PMC5844862 29523813

[8] Ross JS, Sheehan CE, Fisher HA, Kaufman RP Jr, Kaur P, Gray K, et al. Correlation of primary tumor prostate-specific membrane antigen expression with disease recurrence in prostate cancer. Clin Cancer Res. 2003;9(17):6357–62. 14695135

[9] Malaspina S, Anttinen M, Taimen P, Jambor I, Sandell M, Rinta-Kiikka I, et al. Prospective comparison of 18F-PSMA-1007 PET/CT, whole-body MRI and CT in primary nodal staging of unfavourable intermediate- and high-risk prostate cancer. Eur J Nucl Med Mol Imaging. 2021. 10.1007/S00259-021-05296-1 10.1007/s00259-021-05296-1 PMC8263440 33715033

[10] Anttinen M, Ettala O, Malaspina S, Jambor I, Sandell M, Kajander S, et al. A prospective comparison of ^18^F-prostate-specific membrane antigen-1007 positron emission tomography computed tomography, whole-body 1.5 T magnetic resonance imaging with diffusion-weighted imaging, and single-photon emission computed tomography/computed tomography with traditional imaging in primary distant metastasis staging of prostate cancer (PROSTAGE). Eur Urol Oncol. 2021. 10.1016/J.EUO.2020.06.012 10.1016/j.euo.2020.06.012 32675047

[11] Mottet N, Bellmunt J, Bolla M, Briers E, Cumberbatch MG, De Santis M, et al. EAU-ESTRO-SIOG Guidelines on prostate cancer. Part 1: screening, diagnosis, and local treatment with curative intent. Eur Urol. 2017. 10.1016/J.EURURO.2016.08.003 10.1016/j.eururo.2016.08.003 27568654

[12] Fendler WP, Eiber M, Beheshti M, Bomanji J, Calais J, Ceci F, et al. PSMA PET/CT: joint EANM procedure guideline/ SNMM procedure standard for prostate cancer imaging 2.0. Eur J Nucl Med Mol Imaging. 2023. 10.1007/S00259-022-06089-W 10.1007/s00259-022-06089-w PMC10027805 36604326

[13] van Leenders GJLH, van der Kwast TH, Grignon DJ, Evans AJ, Kristiansen G, Kweldam CF, et al. The 2019 International Society of Urological Pathology (ISUP) consensus conference on grading of prostatic carcinoma. Am J Surg Pathol. 2020. 10.1097/PAS.0000000000001497 10.1097/PAS.0000000000001497 PMC7382533 32459716

[14] Bankhead P, Loughrey MB, Fernández JA, Dombrowski Y, McArt DG, Dunne PD, et al. QuPath: open source software for digital pathology image analysis. Sci Rep. 2017. 10.1038/S41598-017-17204-5 10.1038/s41598-017-17204-5 PMC5715110 29203879

[15] Schned AR, Wheeler KJ, Hodorowski CA, Heaney JA, Ernstoff MS, Amdur RJ, et al. Tissue-shrinkage correction factor in the calculation of prostate cancer volume. Am J Surg Pathol. 1996. 10.1097/00000478-199612000-00009 10.1097/00000478-199612000-00009 8944043

[16] Kuten J, Fahoum I, Savin Z, Shamni O, Gitstein G, Hershkovitz D, et al. Head-to-head comparison of 68Ga-PSMA-11 with ^18^F-PSMA-1007 PET/CT in staging prostate cancer using histopathology and immunohistochemical analysis as a reference standard. J Nucl Med. 2020. 10.2967/JNUMED.119.234187 10.2967/jnumed.119.234187 31562225

[17] Luo L, Zheng A, Chang R, Li Y, Gao J, Wang Z, et al. Evaluating the value of ^18^F-PSMA-1007 PET/CT in the detection and identification of prostate cancer using histopathology as the standard. Cancer Imaging. 2023. 10.1186/S40644-023-00627-X 10.1186/s40644-023-00627-x PMC10623763 37924154

[18] Mookerji N, Pfanner T, Hui A, Huang G, Albers P, Mittal R, et al. Fluorine-18 prostate-specific membrane Antigen–1007 PET/CT vs multiparametric MRI for locoregional staging of prostate cancer. JAMA Oncol. 2024. 10.1001/JAMAONCOL.2024.3196 10.1001/jamaoncol.2024.3196 PMC11217889 38949926

[19] Trägårdh E, Simoulis A, Bjartell A, Jögi J. Tumor detection of ^18^F-PSMA-1007 in the prostate gland in patients with prostate cancer using prostatectomy specimens as reference method. J Nucl Med. 2021. 10.2967/JNUMED.121.261993 10.2967/jnumed.121.261993 PMC8612187 33789930

[20] Kesch C, Vinsensia M, Radtke JP, Schlemmer HP, Heller M, Ellert E, et al. Intraindividual comparison of ^18^F-PSMA-1007 PET/CT, multiparametric MRI, and radical prostatectomy specimens in patients with primary prostate cancer: a retrospective, proof-of-concept study. J Nucl Med. 2017. 10.2967/JNUMED.116.189233 10.2967/jnumed.116.189233 28473595

[21] Exterkate L, Hermsen R, Küsters-Vandevelde HVN, Prette JF, Baas DJH, Somford DM, et al. Head-to-head comparison of ^18^F-PSMA-1007 positron emission tomography/computed tomography and multiparametric magnetic resonance imaging with whole-mount histopathology as reference in localisation and staging of primary prostate cancer. Eur Urol Oncol. 2023. 10.1016/J.EUO.2023.04.006 10.1016/j.euo.2023.04.006 37230883

[22] Emmett L, Papa N, Buteau J, Ho B, Liu V, Roberts M, et al. The PRIMARY score: using intraprostatic 68Ga-PSMA PET/CT patterns to optimize prostate cancer diagnosis. J Nucl Med. 2022. 10.2967/JNUMED.121.263448 10.2967/jnumed.121.263448 PMC9635676 35301240

[23] Emmett L, Papa N, Counter W, Calais J, Barbato F, Burger I, et al. Reproducibility and accuracy of the PRIMARY Score on PSMA PET and of PI-RADS on multiparametric MRI for prostate cancer diagnosis within a real-world database. J Nucl Med. 2024. 10.2967/JNUMED.123.266164 10.2967/jnumed.123.266164 38050155

[24] Privé BM, Israël B, Schilham MGM, Muselaers CHJ, Zámecnik P, Mulders PFA, et al. Evaluating F-18-PSMA-1007-PET in primary prostate cancer and comparing it to multi-parametric MRI and histopathology. Prostate Cancer Prostatic Dis. 2021. 10.1038/S41391-020-00292-2 10.1038/s41391-020-00292-2 32999466

[25] Woythal N, Arsenic R, Kempkensteffen C, Miller K, Janssen JC, Huang K, et al. Immunohistochemical validation of PSMA expression measured by 68Ga-PSMA PET/CT in primary prostate cancer. J Nucl Med. 2018. 10.2967/JNUMED.117.195172 10.2967/jnumed.117.195172 28775203

[26] Heetman JG, Hermsen R, Exterkate L, Küsters-Vandevelde HVN, Brouwer LJM, Somford DM, et al. Immunohistochemical and histopathological validation of 18F-PSMA-1007 PET/CT for intraprostatic cancerous lesions. Prostate. 2023. 10.1002/PROS.24595 10.1002/pros.24595 37455399

[27] Cytawa W, Kircher S, Kübler H, Werner RA, Weber S, Hartrampf P, et al. Diverse PSMA expression in primary prostate cancer: reason for negative [68Ga]Ga-PSMA PET/CT scans? Immunohistochemical validation in 40 surgical specimens. Eur J Nucl Med Mol Imaging. 2022. 10.1007/S00259-022-05831-8 10.1007/s00259-022-05831-8 35556160

[28] Rüschoff JH, Ferraro DA, Muehlematter UJ, Laudicella R, Hermanns T, Rodewald AK, et al. What’s behind 68Ga-PSMA-11 uptake in primary prostate cancer PET? Investigation of histopathological parameters and immunohistochemical PSMA expression patterns. Eur J Nucl Med Mol Imaging. 2021. 10.1007/S00259-021-05501-1 10.1007/s00259-021-05501-1 PMC8484204 34386839

[29] Al Jalali V, Wasinger G, Rasul S, Grubmüller B, Wulkersdorfer B, Balber T, et al. Consecutive prostate-specific membrane antigen (PSMA) and antigen receptor (AR) PET imaging shows positive correlation with AR and PSMA protein expression in primary hormone-naïve prostate cancer. J Nucl Med. 2023. 10.2967/JNUMED.122.264981 10.2967/jnumed.122.264981 36657982

[30] Patel KR, van der Heide UA, Kerkmeijer LGW, Schoots IG, Turkbey B, Citrin DE, et al. Target volume optimization for localized prostate cancer. Pract Radiat Oncol. 2024. 10.1016/J.PRRO.2024.06.006 10.1016/j.prro.2024.06.006 PMC11531394 39019208

[31] Spohn SKB, Kramer M, Kiefer S, Bronsert P, Sigle A, Schultze-Seemann W, et al. Comparison of manual and Semi-Automatic [^18^F]PSMA-1007 PET based contouring techniques for intraprostatic tumor delineation in patients with primary prostate cancer and validation with histopathology as standard of reference. Front Oncol. 2020. 10.3389/FONC.2020.600690 10.3389/fonc.2020.600690 PMC7750498 33365271

[32] Draulans C, De Roover R, van der Heide UA, Kerkmeijer L, Smeenk RJ, Pos F, et al. Optimal 68Ga-PSMA and 18F-PSMA PET window levelling for gross tumour volume delineation in primary prostate cancer. Eur J Nucl Med Mol Imaging. 2021. 10.1007/S00259-020-05059-4 10.1007/s00259-020-05059-4 33025093

