# Supplementary material for: Prospective comparison of 18F-PSMA-1007 PET/CT and MRI with histopathology as the reference standard for intraprostatic tumour detection and T-staging of high-risk prostate cancer
Source: Eur J Nucl Med Mol Imaging. 2025 Mar 31;52(10):3709–19. doi: 10.1007/s00259-025-07208-z (PMC12316704; doi:10.1007/s00259-025-07208-z)
Supplement: Supplementary file 1 — Supplementary Material 1 [file 259_2025_7208_MOESM1_ESM.docx]

**Fig S1** A demonstration of the methodology used in the diameter analysis. **(a)** PSMA PET image showing an index lesion delineated with the threshold SUVbackground x2 (blue). The green circle represents constraint around the prostate gland. The lesion measured 11 mm in transaxial diameter. **(b)** HE-stained cross section of the prostate with the corresponding index lesion annotated. The lesion measured 11 mm in transaxial diameter. Scale bar 5 mm

**
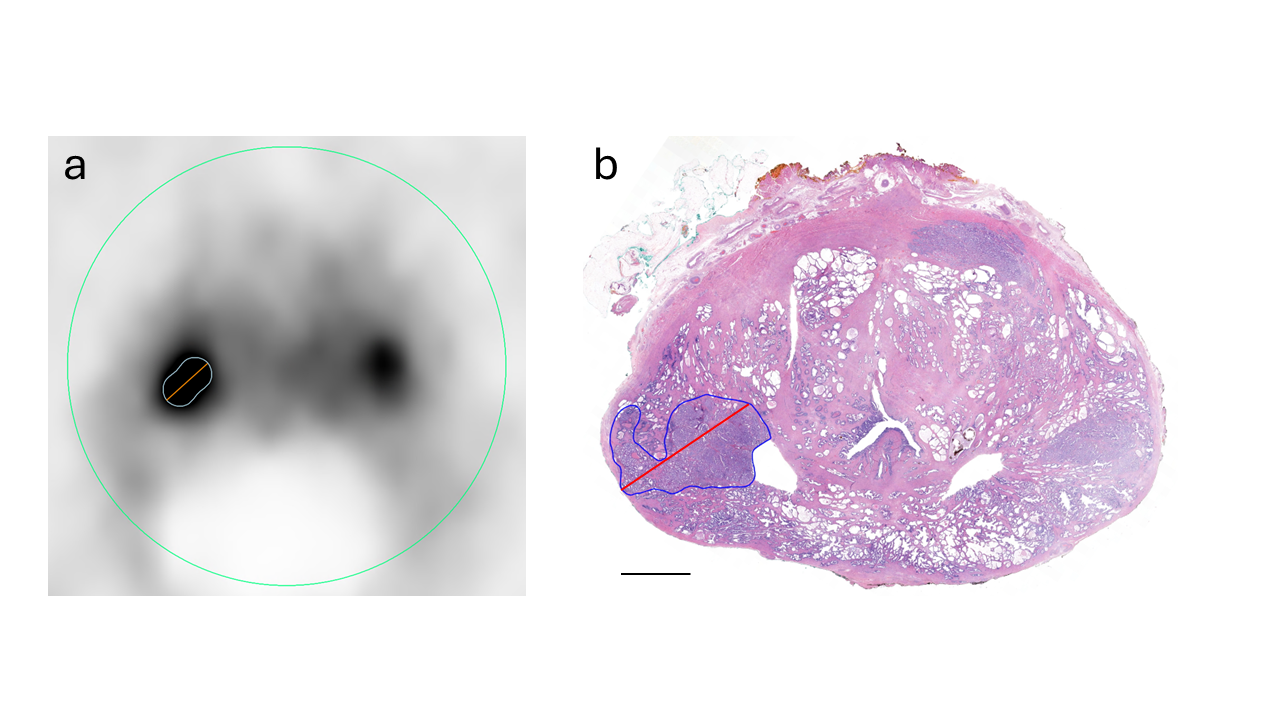
**

**Fig. S2** The only case of SVI detected by MRI but undetected by PET/CT. The diameter of the intravesicular lesion in histopathology was 2,4 mm. **(a)** Fat-suppressed axial T2 image demonstrating SVI on the left (arrow). **(b)** Axial DWI image (b-value of 1500 s/mm2) demonstrating SVI on the left (arrow). **(c)** PET/CT image showing no uptake in the left seminal vesicle (circle)


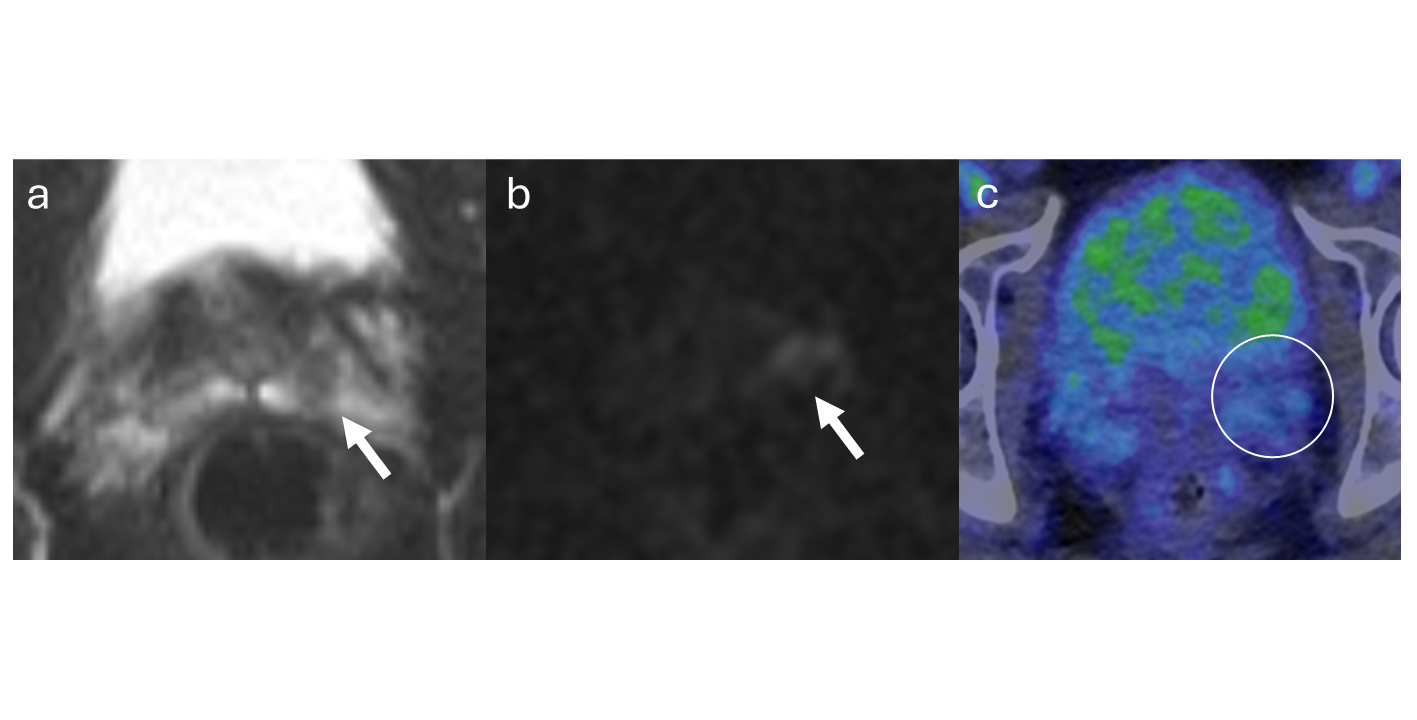


**Table S1** Detailed characteristics of all the histological lesions and the two false-positive lesions detected by PSMA PET/CT reader 2. The pairing of the lesions is according to the consensus reading. The false-positive and false-negative lesions are highlighted with light grey colour

| **Patient** | **Lesion number** | **Histological location** | **Diameter (mm)** | **Mean ODmax of cytoplasmic DAB** | **Gleason score** | **SUVmax** |
| --- | --- | --- | --- | --- | --- | --- |
| 1 | 1 | BLP, MLP | 13 | 1.442 | 4+5 | 24.9 |
| 2 | 1 | BRP, BLP, MRP | 11 | 1.177 | 4+5 | 51.7 |
| 3 | 1 | MRA, MRP | 6 | 2.522 | 4+4 | 14.3 |
| 3 | 2 | MLP, ALP | 5 | 1.924 | 4+3 | 21.8 |
| 3 | 3 | MLA | 6 | 0.377 | 3+3 | 21.8 |
| 4 | 1 | MLP, ALP | 14 | 0.758 | 4+4 | 7.6 |
| 4 | 2 | MRA, MLA, ARA, ALA | 22 | 0.909 | 3+4 | 10.4 |
| 5 | 1 | MLP, ALP | 13 | 2.021 | 4+3+5 | 22.0 |
| 5 | 2 | ARP | 5 | 1.278 | 3+4 | 5.3 |
| 6 | 1 | MLP | 9 | 2.123 | 4+5 | 6.3 |
| 6 | 2 | MLA, BLA | 14 | 0.669 | 3+4 | 6.3 |
| 6 | 3 | BRA, MRA | 18 | 0.624 | 3+4 | 5.7 |
| 6 | 4 | ARA, ARP | 4 | 1.866 | 3+3 | 5.5 |
| 7 | 1 | MRP, ARP | 11 | 1.164 | 4+4 | 9.6 |
| 7 | 2 | MLP | 7 | 1.838 | 4+3 | 5.8 |
| 7 | 3 | MLA | 10 | 0.241 | 3+4 | N/A |
| 8 | 1 | MLA, ALA | 20 | 1.193 | 3+4+5 | 8.5 |
| 8 | 2 | MRP | 15 | 0.855 | 3+4 | 5.4 |
| 8 | 3 | MRA | 9 | 1.434 | 3+3+4 | 5.4 |
| 9 | 1 | MLP, ALP | 19 | 2.123 | 4+3+5 | 12.0 |
| 9 | 2 | MRP | 4 | 0.278 | 3+3 | N/A |
| 9 | N/A | N/A | N/A | N/A | N/A | 6.5 |
| 10 | 1 | MRA, MLA | 21 | 1.227 | 4+3+5 | 43.2 |
| 10 | 2 | MRP | 7 | 0.309 | 3+4 | N/A |
| 11 | 1 | BLP, MLP | 24 | 1.737 | 4+5 | 22.2 |
| 11 | 2 | MRA, MLA, ARA, ALA | 9 | 0.284 | 3+3 | N/A |
| 12 | 1 | BRP, BLA, BLP, MLA, MLP, ALA, ALP | 39 | 0.347 | 4+5 | 7.0 |
| 13 | 1 | MRP, ARP | 24 | 1.467 | 4+3 | 20.7 |
| 13 | 2 | BRA, MRA | 14 | 0.553 | 3+4 | 9.3 |
| 13 | 3 | MLA | 16 | 0.667 | 3+4 | 7.4 |
| 14 | 1 | BRP, BLP, MRA, MRP, MLA, MLP, ARA, ARP, ALP | 37 | 1.276 | 4+5 | 15.7 |
| 15 | 1 | ALA, ALP | 17 | 0.976 | 4+3+5 | 27.1 |
| 16 | 1 | MRA, ARA | 14 | 0.670 | 3+5 | 18.8 |
| 16 | 2 | MLA, ALA | 10 | 0.293 | 3+4 | 5.0 |
| 17 | 1 | MRA | 10 | 1.962 | 4+4 | 15.9 |
| 17 | N/A | N/A | N/A | N/A | N/A | 4.9 |
| 18 | 1 | BRP, BLP, MRP, MLP, ARP, ALP | 42 | 0.772 | 4+3 | 7.1 |
| 18 | 2 | MRA, MLA, ARA, ALA | 15 | 0.484 | 3+3 | N/A |
| 19 | 1 | MRP, ARP | 18 | 0.596 | 3+4 | 19.1 |
| 19 | 2 | MLP | 10 | 0.562 | 3+4 | 6.4 |
| 19 | 3 | ARA, ALA | 17 | 0.653 | 3+3 | N/A |

ODmax = maximum optical density, DAB = 3,3'-diaminobenzidine, SUVmax = maximum standardized uptake value. Lesion number 1 indicates the index lesion. For histological location, the positive segments for each lesion are reported with three letters; first either B (basis), M (midprostate) or A (apex), second either R (right) or L (left) and third either A (anterior) or P (posterior)

**Fig. S3** False-negative lesions. For each lesion, a low magnification H&E image, a high magnification PSMA IHC image and a fused PET/CT image are presented. In PET/CT images, the area in which the lesion should have appeared is marked with a circle. The detailed characteristics of each lesion are represented in Table S1. Images a-c, d-f, g-i, j-l, m-o and p-r are of patients 7, 9, 10, 11, 18 and 19, respectively. Scale bars 5 mm (a, d, g, j, m, p), 1 mm (b, h, k, n, q) and 0,5 mm (e)

**
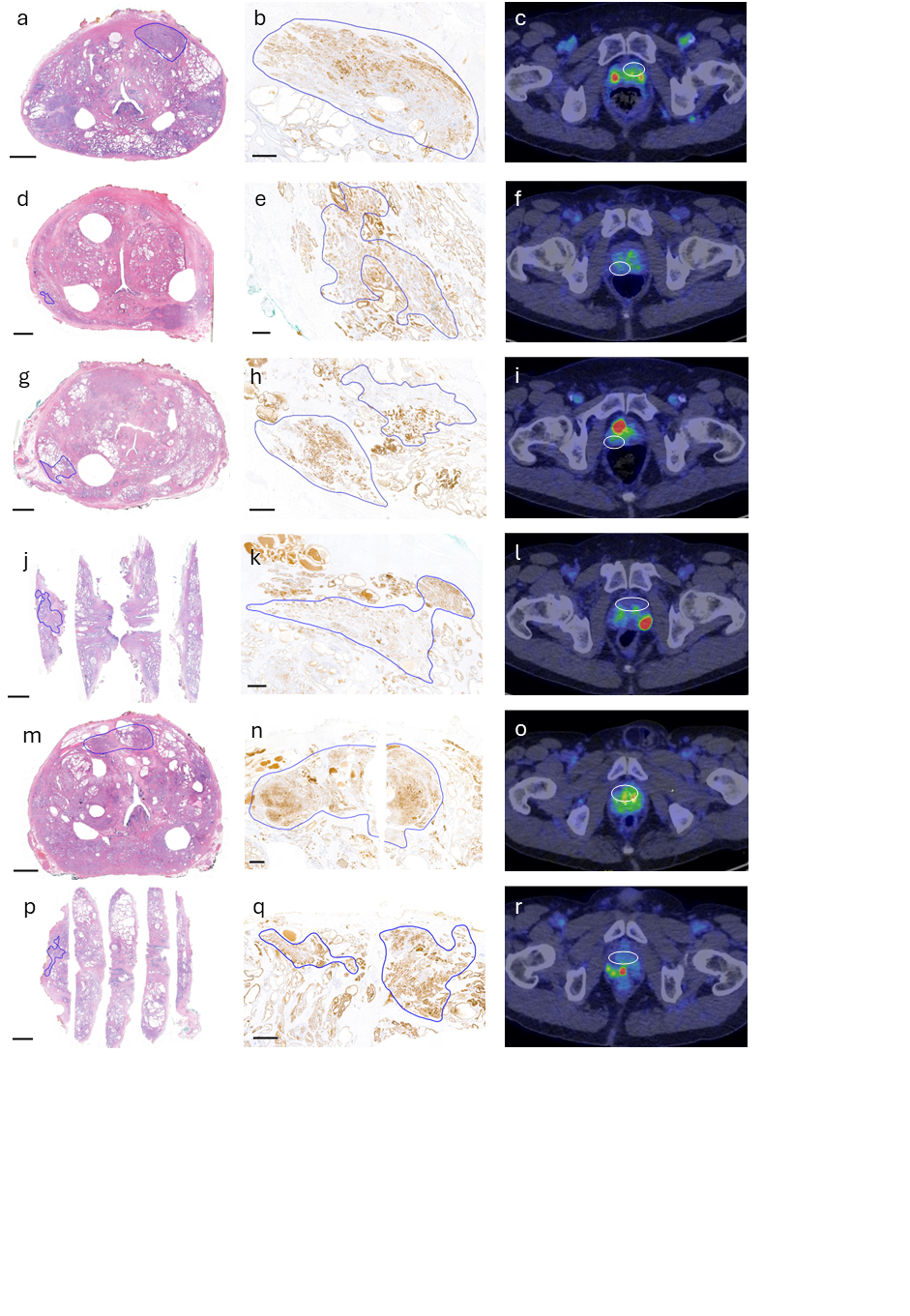
**

**Fig. S4** False-positive lesions. For each lesion, a low magnification H&E image, a high magnification PSMA IHC image and a fused PET/CT image are presented. The detailed characteristics of each lesion are represented in Table S1. Images a-c are of patient 9, and images d-f are of patient 17. Scale bars 5 mm (a-b, d-e)


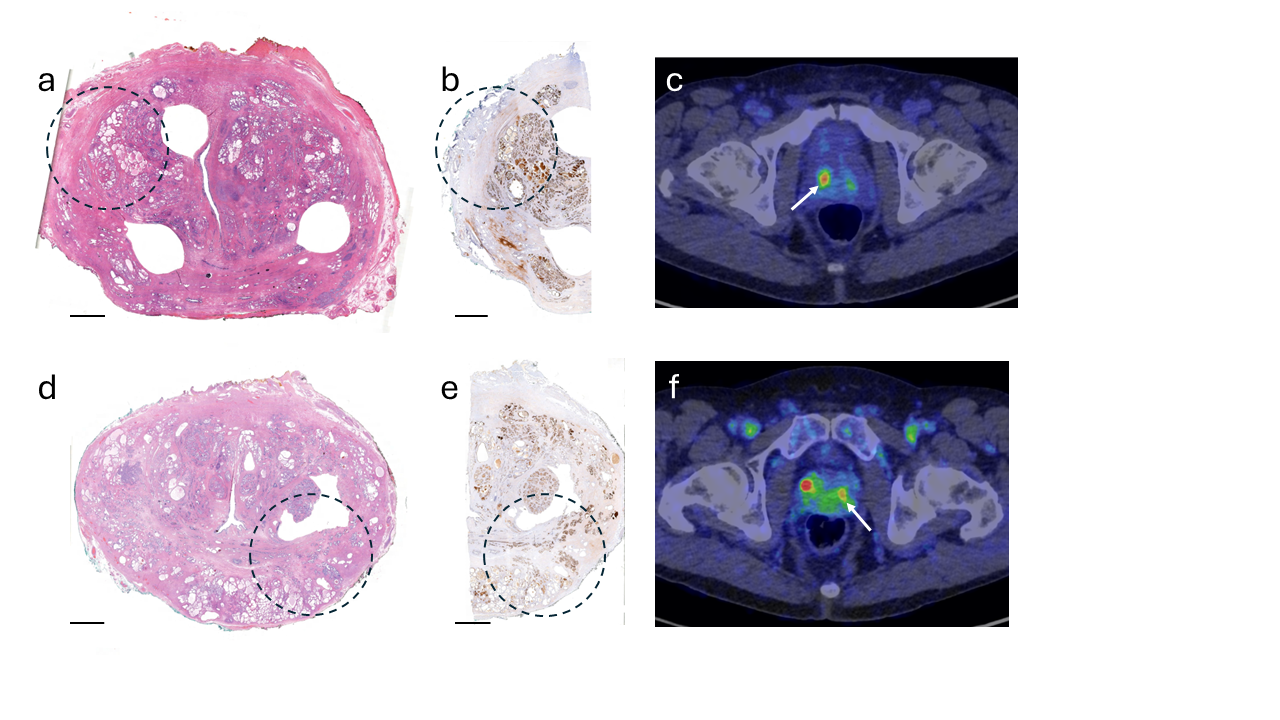


**Fig. S5** A scatter plot demonstrating the correlation between SUVmax and cytoplasmic DAB ODmax multiplied by lesion diameter


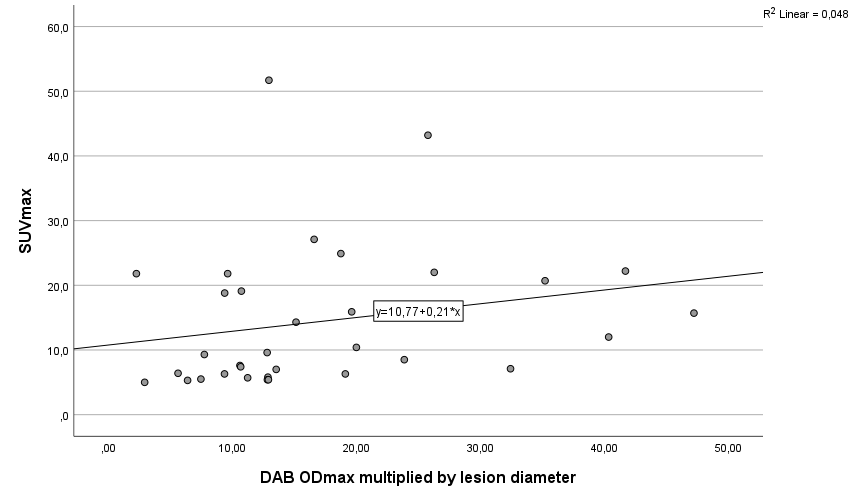


SUVmax = maximum standardized uptake value, ODmax = maximum optical density, DAB = 3,3'-diaminobenzidine
